# 2-[5-(4-Hydroxy­phen­yl)-1-phenyl-1*H*-pyrazol-3-yl]phenol

**DOI:** 10.1107/S1600536808017054

**Published:** 2008-06-13

**Authors:** Amir Badshah, Aurangzeb Hasan, Cecilia R. Barbarín, Mehwash Zia

**Affiliations:** a Department of Chemistry, Quaid-i-Azam University, Islamabad 45320, Pakistan; b División de Estudios de Posgrado, Facultad de Ciencias Químicas, U.A.N.L., Guerreo y Progreso S/N, Col. Treviño, C.P. 64570, Monterrey, NL, Mexico

## Abstract

The title compound, C_21_H_16_N_2_O_2_, was derived from 1-(2-hydroxy­phen­yl)-3-(4-methoxy­phen­yl)propane-1,3-dione. The pyrazole ring and one of the hydr­oxy-substituted benzene rings are approximately coplanar, forming a dihedral angle of 7.5 (3)°. The relative conformation of these rings may be influenced by an intra­molecular O—H⋯N hydrogen bond. In the crystal structure, inter­molecular O—H⋯O hydrogen bonds involving different hydr­oxy groups of symmetry-related mol­ecules form extended chains along [201].

## Related literature

For related literature, see: Ahmad *et al.* (1990 ▶, 1997 ▶); Beeam *et al.* (1984 ▶); Elguero (1983 ▶); Trofinenko (1972 ▶).
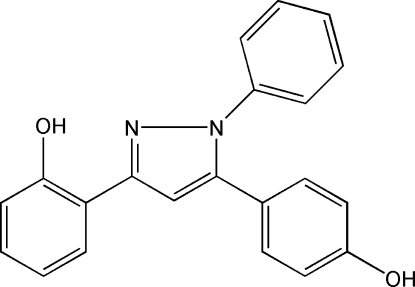


## Experimental

### 

#### Crystal data

C_21_H_16_N_2_O_2_
*M* *_r_* = 328.36
Monoclinic, 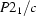

*a* = 10.793 (3) Å
*b* = 12.948 (3) Å
*c* = 11.705 (3) Å
β = 93.508 (14)°
*V* = 1632.7 (7) Å^3^
*Z* = 4
Mo *K*α radiation
μ = 0.09 mm^−1^
*T* = 298 (2) K
0.44 × 0.40 × 0.26 mm

#### Data collection

Siemens P4 diffractometer
Absorption correction: none
5767 measured reflections
3720 independent reflections
2353 reflections with *I* > 2σ(*I*)
*R*_int_ = 0.024
3 standard reflections every 97 reflections intensity decay: 3.6%

#### Refinement

*R*[*F* ^2^ > 2σ(*F* ^2^)] = 0.047
*wR*(*F* ^2^) = 0.128
*S* = 1.03
3720 reflections
227 parameters
H-atom parameters constrained
Δρ_max_ = 0.15 e Å^−3^
Δρ_min_ = −0.16 e Å^−3^

### 

Data collection: *XSCANS* (Siemens, 1999 ▶); cell refinement: *XSCANS*; data reduction: *XSCANS*; program(s) used to solve structure: *SHELXTL-Plus* (Sheldrick, 2008 ▶); program(s) used to refine structure: *SHELXTL-Plus*; molecular graphics: *SHELXTL-Plus* and *Mercury* (Macrae *et al*., 2006); software used to prepare material for publication: *SHELXTL-Plus*.

## Supplementary Material

Crystal structure: contains datablocks I, global. DOI: 10.1107/S1600536808017054/lh2632sup1.cif

Structure factors: contains datablocks I. DOI: 10.1107/S1600536808017054/lh2632Isup2.hkl

Additional supplementary materials:  crystallographic information; 3D view; checkCIF report

## Figures and Tables

**Table 1:** Hydrogen-bond geometry (Å, °)

*D*—H⋯*A*	*D*—H	H⋯*A*	*D*⋯*A*	*D*—H⋯*A*
O1—H1*B*⋯O2^i^	0.82	2.05	2.824 (2)	158
O2—H2*B*⋯N2	0.82	1.87	2.595 (2)	147

Symmetry code: (i) 
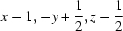
.

**Fig. 1.**
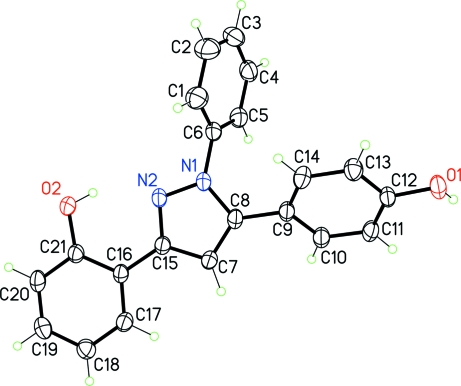
The molecular structure of (III) showing the atom-labeling scheme. Displacement ellipsoids are drawn at the 30% probability level and H atoms are shown as small spheres of arbitrary radii.

**Fig. 2.**
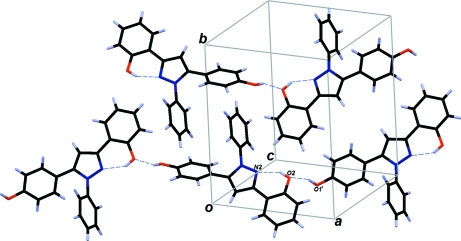
Part of the crystal structure of (III) showing the hydrogen bonds as dashed lines.

**Fig. 3.**
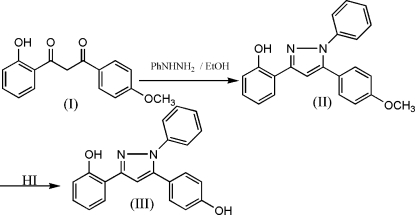
Reaction scheme.

## supplementary crystallographic information

### Comment

Pyrazoles are important because of their potential for biological activity (Beeam *et al.*, 1984). Both traditional and new scientific methods have been used used to prepare new materials for medicine (Elguero *et al.*, 1983) and agriculture (Trofinenko, 1972). Neutral and anionic pyrazoles are excellent ligands and their co-ordination chemistry has been extensively studied (Bonati, 1980). In the molecular structure of the title compound (III) (Fig. 1 and Fig. 3) there is an intramolecular hydrogen bond between the OH group of one phenolic group and the N atom of the pyrazole group (see Table 1 for hydrogen bond details). One of the phenyl groups is approximately coplanar with the pyrazole groups (dihedral angle = 7.5 (3)°), possibly due to the intramolecular hydrogen bond formation. The other two phenyl groups are rotated by 66.4 (12)°. In the crystal structure an intermolecular hydrogen bond between non equivalent hydroxy groups of symmetry related molecules, forms extended chains along [201] (Fig. 2).

### Experimental

Compound (I) [see Fig. 3] was prepared by a modified Baker Venkataram rearrangement as reported earlier (Ahmad *et al.*, 1990, 1997). Purification was carried out by recrystallization using absolute ethanol. Compound (II) was synthesized by adding 0.1 mole of phenyl hydrazine in 0.1 mole of compound (II) dissolved in 200 ml of absolute ethanol. The mixture was refluxed for 7 h. Solvent was removed under reduced pressure. Highly viscous residue was recrystallized using absolute ethanol. Compound (III) was synthesized by demethylation of compound (II) using 48% hydrogen bromide in acetic acid. Single crystals suitable for X-ray analysis were obtained by recrystallization from an ethanol solution of (III) at room temperature (Yield: 96%, m.p: 490K).

### Refinement

All H atoms were placed in idealized positions and treated as riding atoms, with C—H = 0.93Å, O-H = 0.82Å and *U*_iso_(H) = 1.2*U*_eq_(C) or 1.5*U*_eq_(O).

### Crystal data

**Table:**

C_21_H_16_N_2_O_2_	*F*_000_ = 688
*M**_r_* = 328.36	*D*_x_ = 1.336 Mg m^−^^3^
Monoclinic, *P*2_1_/*c*	Melting point: 490 K
Hall symbol: -P 2ybc	Mo *K*α radiation λ = 0.71073 Å
*a* = 10.793 (3) Å	Cell parameters from 84 reflections
*b* = 12.948 (3) Å	θ = 4.6–12.4º
*c* = 11.705 (3) Å	µ = 0.09 mm^−^^1^
β = 93.508 (14)º	*T* = 298 (2) K
*V* = 1632.7 (7) Å^3^	Prismatic, colourless
*Z* = 4	0.44 × 0.40 × 0.26 mm

### Data collection

**Table:**

Siemens P4 diffractometer	*R*_int_ = 0.024
Radiation source: fine-focus sealed tube	θ_max_ = 27.5º
Monochromator: graphite	θ_min_ = 1.9º
*T* = 298(2) K	*h* = −14→4
2θ/ω scans	*k* = −16→1
Absorption correction: none	*l* = −15→15
5767 measured reflections	3 standard reflections
3720 independent reflections	every 97 reflections
2353 reflections with *I* > 2σ(*I*)	intensity decay: 3.7%

### Refinement

**Table:**

Refinement on *F*^2^	Hydrogen site location: inferred from neighbouring sites
Least-squares matrix: full	H-atom parameters constrained
*R*[*F*^2^ > 2σ(*F*^2^)] = 0.047	*w* = 1/[σ^2^(*F*_o_^2^) + (0.0468*P*)^2^ + 0.4416*P*] where *P* = (*F*_o_^2^ + 2*F*_c_^2^)/3
*wR*(*F*^2^) = 0.128	(Δ/σ)_max_ < 0.001
*S* = 1.03	Δρ_max_ = 0.15 e Å^−^^3^
3720 reflections	Δρ_min_ = −0.16 e Å^−^^3^
227 parameters	Extinction correction: SHELXTL-Plus (Sheldrick, 2008), Fc^*^=kFc[1+0.001xFc^2^λ^3^/sin(2θ)]^-1/4^
Primary atom site location: structure-invariant direct methods	Extinction coefficient: 0.0155 (18)
Secondary atom site location: difference Fourier map	

### Special details

**Table:**

Geometry. All e.s.d.'s (except the e.s.d. in the dihedral angle between two l.s. planes) are estimated using the full covariance matrix. The cell e.s.d.'s are taken into account individually in the estimation of e.s.d.'s in distances, angles and torsion angles; correlations between e.s.d.'s in cell parameters are only used when they are defined by crystal symmetry. An approximate (isotropic) treatment of cell e.s.d.'s is used for estimating e.s.d.'s involving l.s. planes.

### Fractional atomic coordinates and isotropic or equivalent isotropic displacement parameters (Å^2^)

**Table:**

	*x*	*y*	*z*	*U*_iso_*/*U*_eq_	
O1	−0.19429 (12)	0.33852 (11)	−0.38082 (11)	0.0598 (4)	
H1B	−0.2627	0.3197	−0.3621	0.090*	
O2	0.55378 (12)	0.16872 (10)	0.17109 (13)	0.0603 (4)	
H2B	0.5054	0.2064	0.1339	0.090*	
N1	0.26395 (13)	0.27005 (12)	−0.01205 (13)	0.0467 (4)	
N2	0.34918 (13)	0.21948 (12)	0.05691 (13)	0.0462 (4)	
C1	0.3431 (2)	0.43942 (17)	−0.05478 (19)	0.0628 (6)	
H1A	0.4075	0.4086	−0.0915	0.075*	
C2	0.3343 (3)	0.54518 (19)	−0.0493 (2)	0.0775 (7)	
H2A	0.3933	0.5863	−0.0821	0.093*	
C3	0.2391 (2)	0.58993 (18)	0.0041 (2)	0.0721 (7)	
H3A	0.2326	0.6615	0.0064	0.087*	
C4	0.1531 (2)	0.53006 (17)	0.0543 (2)	0.0653 (6)	
H4A	0.0891	0.5610	0.0915	0.078*	
C5	0.16142 (18)	0.42387 (16)	0.04968 (17)	0.0546 (5)	
H5A	0.1035	0.3827	0.0837	0.066*	
C6	0.25650 (16)	0.38005 (14)	−0.00589 (15)	0.0460 (4)	
C7	0.21875 (16)	0.10653 (14)	−0.03158 (15)	0.0437 (4)	
H7A	0.1815	0.0444	−0.0540	0.052*	
C8	0.18333 (15)	0.20293 (15)	−0.06634 (15)	0.0433 (4)	
C9	0.08425 (15)	0.23722 (14)	−0.14967 (15)	0.0441 (4)	
C10	−0.03190 (17)	0.19422 (15)	−0.15016 (17)	0.0523 (5)	
H10A	−0.0474	0.1428	−0.0975	0.063*	
C11	−0.12620 (17)	0.22564 (16)	−0.22714 (17)	0.0543 (5)	
H11A	−0.2041	0.1950	−0.2266	0.065*	
C12	−0.10491 (16)	0.30191 (14)	−0.30416 (15)	0.0460 (4)	
C13	0.01140 (17)	0.34379 (16)	−0.30736 (16)	0.0516 (5)	
H13A	0.0269	0.3941	−0.3614	0.062*	
C14	0.10531 (17)	0.31154 (16)	−0.23075 (16)	0.0507 (5)	
H14A	0.1841	0.3402	−0.2337	0.061*	
C15	0.32262 (15)	0.11962 (14)	0.04482 (14)	0.0407 (4)	
C16	0.40064 (15)	0.04285 (14)	0.10482 (14)	0.0404 (4)	
C17	0.36890 (17)	−0.06076 (15)	0.10014 (15)	0.0476 (4)	
H17A	0.2955	−0.0804	0.0603	0.057*	
C18	0.44262 (18)	−0.13518 (16)	0.15259 (17)	0.0554 (5)	
H18A	0.4201	−0.2044	0.1471	0.066*	
C19	0.55033 (18)	−0.10638 (17)	0.21343 (16)	0.0546 (5)	
H19A	0.6000	−0.1563	0.2503	0.066*	
C20	0.58441 (18)	−0.00551 (16)	0.21988 (16)	0.0525 (5)	
H20A	0.6572	0.0133	0.2613	0.063*	
C21	0.51143 (16)	0.06923 (14)	0.16520 (15)	0.0447 (4)	

### Atomic displacement parameters (Å^2^)

**Table:**

	*U*^11^	*U*^22^	*U*^33^	*U*^12^	*U*^13^	*U*^23^
O1	0.0470 (8)	0.0663 (9)	0.0638 (8)	0.0073 (7)	−0.0160 (6)	0.0117 (7)
O2	0.0474 (8)	0.0503 (8)	0.0796 (10)	0.0043 (6)	−0.0253 (7)	−0.0134 (7)
N1	0.0380 (8)	0.0451 (9)	0.0548 (9)	0.0054 (7)	−0.0141 (7)	−0.0037 (7)
N2	0.0375 (8)	0.0480 (9)	0.0514 (8)	0.0067 (7)	−0.0119 (7)	−0.0054 (7)
C1	0.0587 (13)	0.0636 (14)	0.0668 (13)	−0.0021 (11)	0.0084 (10)	0.0014 (11)
C2	0.0867 (18)	0.0632 (15)	0.0824 (16)	−0.0137 (14)	0.0036 (14)	0.0141 (13)
C3	0.0878 (18)	0.0479 (12)	0.0775 (15)	0.0045 (12)	−0.0202 (14)	0.0046 (11)
C4	0.0574 (13)	0.0601 (13)	0.0764 (14)	0.0163 (11)	−0.0138 (11)	−0.0140 (11)
C5	0.0446 (10)	0.0554 (12)	0.0631 (12)	0.0036 (9)	−0.0028 (9)	−0.0045 (10)
C6	0.0423 (10)	0.0457 (10)	0.0483 (10)	0.0043 (8)	−0.0104 (8)	−0.0023 (8)
C7	0.0368 (9)	0.0468 (10)	0.0467 (9)	−0.0006 (8)	−0.0051 (7)	−0.0039 (8)
C8	0.0324 (8)	0.0524 (10)	0.0443 (9)	0.0035 (8)	−0.0032 (7)	−0.0031 (8)
C9	0.0348 (9)	0.0504 (10)	0.0460 (9)	0.0036 (8)	−0.0063 (7)	−0.0021 (8)
C10	0.0423 (10)	0.0558 (11)	0.0572 (11)	−0.0031 (9)	−0.0089 (8)	0.0118 (9)
C11	0.0363 (9)	0.0598 (12)	0.0651 (12)	−0.0072 (9)	−0.0108 (9)	0.0073 (10)
C12	0.0400 (9)	0.0490 (10)	0.0475 (10)	0.0079 (8)	−0.0103 (8)	−0.0008 (8)
C13	0.0477 (11)	0.0601 (12)	0.0465 (10)	−0.0009 (9)	−0.0014 (8)	0.0095 (9)
C14	0.0359 (9)	0.0654 (12)	0.0500 (10)	−0.0050 (9)	−0.0021 (8)	0.0021 (9)
C15	0.0351 (9)	0.0465 (10)	0.0400 (8)	0.0025 (8)	−0.0022 (7)	−0.0052 (8)
C16	0.0344 (8)	0.0487 (10)	0.0375 (8)	0.0031 (8)	−0.0034 (7)	−0.0033 (7)
C17	0.0415 (10)	0.0524 (11)	0.0478 (10)	−0.0047 (8)	−0.0051 (8)	0.0032 (8)
C18	0.0518 (11)	0.0526 (12)	0.0612 (11)	0.0003 (9)	−0.0005 (9)	0.0094 (9)
C19	0.0480 (11)	0.0617 (13)	0.0540 (11)	0.0108 (10)	0.0012 (9)	0.0122 (10)
C20	0.0407 (10)	0.0663 (13)	0.0488 (10)	0.0059 (9)	−0.0097 (8)	−0.0004 (9)
C21	0.0391 (9)	0.0491 (10)	0.0449 (9)	0.0044 (8)	−0.0052 (8)	−0.0084 (8)

### Geometric parameters (Å, °)

**Table:**

O1—C12	1.362 (2)	C8—C9	1.471 (2)
O1—H1B	0.8200	C9—C10	1.371 (3)
O2—C21	1.367 (2)	C9—C14	1.380 (3)
O2—H2B	0.8200	C10—C11	1.379 (3)
N1—N2	1.3549 (19)	C10—H10A	0.9300
N1—C8	1.360 (2)	C11—C12	1.366 (3)
N1—C6	1.428 (2)	C11—H11A	0.9300
N2—C15	1.330 (2)	C12—C13	1.370 (3)
C1—C6	1.363 (3)	C13—C14	1.376 (3)
C1—C2	1.374 (3)	C13—H13A	0.9300
C1—H1A	0.9300	C14—H14A	0.9300
C2—C3	1.364 (4)	C15—C16	1.455 (2)
C2—H2A	0.9300	C16—C17	1.385 (3)
C3—C4	1.369 (3)	C16—C21	1.394 (2)
C3—H3A	0.9300	C17—C18	1.371 (3)
C4—C5	1.379 (3)	C17—H17A	0.9300
C4—H4A	0.9300	C18—C19	1.377 (3)
C5—C6	1.371 (3)	C18—H18A	0.9300
C5—H5A	0.9300	C19—C20	1.358 (3)
C7—C8	1.360 (3)	C19—H19A	0.9300
C7—C15	1.401 (2)	C20—C21	1.380 (3)
C7—H7A	0.9300	C20—H20A	0.9300
			
C12—O1—H1B	109.5	C11—C10—H10A	119.3
C21—O2—H2B	109.5	C12—C11—C10	119.82 (17)
N2—N1—C8	111.18 (15)	C12—C11—H11A	120.1
N2—N1—C6	119.34 (14)	C10—C11—H11A	120.1
C8—N1—C6	128.64 (14)	O1—C12—C11	123.06 (17)
C15—N2—N1	105.81 (13)	O1—C12—C13	117.22 (17)
C6—C1—C2	119.5 (2)	C11—C12—C13	119.71 (16)
C6—C1—H1A	120.3	C12—C13—C14	120.10 (18)
C2—C1—H1A	120.3	C12—C13—H13A	120.0
C3—C2—C1	120.0 (2)	C14—C13—H13A	120.0
C3—C2—H2A	120.0	C13—C14—C9	120.92 (17)
C1—C2—H2A	120.0	C13—C14—H14A	119.5
C2—C3—C4	120.4 (2)	C9—C14—H14A	119.5
C2—C3—H3A	119.8	N2—C15—C7	110.14 (15)
C4—C3—H3A	119.8	N2—C15—C16	119.87 (15)
C3—C4—C5	120.0 (2)	C7—C15—C16	129.96 (16)
C3—C4—H4A	120.0	C17—C16—C21	117.38 (16)
C5—C4—H4A	120.0	C17—C16—C15	120.54 (15)
C6—C5—C4	118.9 (2)	C21—C16—C15	122.04 (16)
C6—C5—H5A	120.5	C18—C17—C16	121.86 (18)
C4—C5—H5A	120.5	C18—C17—H17A	119.1
C1—C6—C5	121.20 (19)	C16—C17—H17A	119.1
C1—C6—N1	119.94 (18)	C17—C18—C19	119.33 (19)
C5—C6—N1	118.86 (18)	C17—C18—H18A	120.3
C8—C7—C15	106.22 (15)	C19—C18—H18A	120.3
C8—C7—H7A	126.9	C20—C19—C18	120.44 (18)
C15—C7—H7A	126.9	C20—C19—H19A	119.8
N1—C8—C7	106.65 (14)	C18—C19—H19A	119.8
N1—C8—C9	122.37 (17)	C19—C20—C21	120.24 (18)
C7—C8—C9	130.90 (17)	C19—C20—H20A	119.9
C10—C9—C14	117.98 (16)	C21—C20—H20A	119.9
C10—C9—C8	120.49 (17)	O2—C21—C20	117.24 (16)
C14—C9—C8	121.51 (16)	O2—C21—C16	122.02 (16)
C9—C10—C11	121.40 (18)	C20—C21—C16	120.73 (18)
C9—C10—H10A	119.3		
			
C8—N1—N2—C15	0.7 (2)	C10—C11—C12—O1	−178.41 (18)
C6—N1—N2—C15	171.10 (16)	C10—C11—C12—C13	2.6 (3)
C6—C1—C2—C3	−0.4 (4)	O1—C12—C13—C14	178.78 (17)
C1—C2—C3—C4	1.2 (4)	C11—C12—C13—C14	−2.2 (3)
C2—C3—C4—C5	−0.9 (3)	C12—C13—C14—C9	−0.2 (3)
C3—C4—C5—C6	−0.1 (3)	C10—C9—C14—C13	2.0 (3)
C2—C1—C6—C5	−0.7 (3)	C8—C9—C14—C13	−179.23 (17)
C2—C1—C6—N1	179.36 (19)	N1—N2—C15—C7	−0.6 (2)
C4—C5—C6—C1	0.9 (3)	N1—N2—C15—C16	177.64 (15)
C4—C5—C6—N1	−179.09 (17)	C8—C7—C15—N2	0.4 (2)
N2—N1—C6—C1	76.2 (2)	C8—C7—C15—C16	−177.66 (17)
C8—N1—C6—C1	−115.2 (2)	N2—C15—C16—C17	175.03 (17)
N2—N1—C6—C5	−103.7 (2)	C7—C15—C16—C17	−7.1 (3)
C8—N1—C6—C5	64.8 (3)	N2—C15—C16—C21	−7.4 (3)
N2—N1—C8—C7	−0.4 (2)	C7—C15—C16—C21	170.51 (18)
C6—N1—C8—C7	−169.74 (17)	C21—C16—C17—C18	0.1 (3)
N2—N1—C8—C9	−177.64 (16)	C15—C16—C17—C18	177.79 (17)
C6—N1—C8—C9	13.1 (3)	C16—C17—C18—C19	1.1 (3)
C15—C7—C8—N1	0.0 (2)	C17—C18—C19—C20	−1.1 (3)
C15—C7—C8—C9	176.91 (18)	C18—C19—C20—C21	−0.1 (3)
N1—C8—C9—C10	−138.9 (2)	C19—C20—C21—O2	−177.28 (18)
C7—C8—C9—C10	44.6 (3)	C19—C20—C21—C16	1.4 (3)
N1—C8—C9—C14	42.4 (3)	C17—C16—C21—O2	177.27 (17)
C7—C8—C9—C14	−134.1 (2)	C15—C16—C21—O2	−0.4 (3)
C14—C9—C10—C11	−1.6 (3)	C17—C16—C21—C20	−1.4 (3)
C8—C9—C10—C11	179.66 (18)	C15—C16—C21—C20	−179.04 (17)
C9—C10—C11—C12	−0.7 (3)		

### Hydrogen-bond geometry (Å, °)

**Table:**

*D*—H···*A*	*D*—H	H···*A*	*D*···*A*	*D*—H···*A*
O1—H1B···O2^i^	0.82	2.05	2.824 (2)	158
O2—H2B···N2	0.82	1.87	2.595 (2)	147

Symmetry codes: (i) *x*−1, −*y*+1/2, *z*−1/2.

## References

[bb1] Ahmad, R., Malik, M. A. & Zia-ul-Haq, M. (1990). *J. Chem. Soc. Pak.* **12**, 352–355.

[bb2] Ahmad, R., Zia-ul-Haq, M., Duddeek, H., Stefaniak, L. & Kowski, J. S. (1997). *Monatsh. Chem.* **128**, 633–640.

[bb3] Beeam, C. F., Hall, H. L., Huff, A. M., Tummons, R. C. & Grady, S. A. O. (1984). *J. Heteroatom. Chem.* **21**, 1897–1902.

[bb4] Bonati, F. (1980). *Chim. Ind. (Roma)*, **62**, 323–328,.

[bb5] Elguero, J. (1983). Comprehensive Heterocyclic Chemistry, Vol. 5, Part 4A, pp. 167, 304. Elmford, New York: Pergamon Press.

[bb9] Macrae, C. F., Edgington, P. R., McCabe, P., Pidcock, E., Shields, G. P., Taylor, R., Towler, M. & van de Streek, J. (2006). *J. Appl. Cryst.* **39**, 453–457.

[bb6] Sheldrick, G. M. (2008). *Acta Cryst.* A**64**, 112–122. 10.1107/S0108767307043930 18156677

[bb7] Siemens (1999). *XSCANS User’s Manual* Siemens Analytical X-Ray Instruments Inc., Madison, Wisconsin, USA.

[bb8] Trofinenko, S. (1972). *Chem. Rev.* **72**, 497–500.

